# Seasonal gene expression kinetics between diapause phases in *Drosophila virilis* group species and overwintering differences between diapausing and non-diapausing females

**DOI:** 10.1038/srep11197

**Published:** 2015-06-11

**Authors:** Tiina S. Salminen, Laura Vesala, Asta Laiho, Mikko Merisalo, Anneli Hoikkala, Maaria Kankare

**Affiliations:** 1 University of Jyvaskyla, Department of Biological and Environmental Science, P.O. Box 35, FI-40014, University of Jyväskylä, Finland; 2 BioMediTech, University of Tampere, Biokatu 6, F1-33014 Finland; 3 Finnish DNA Microarray Centre, Bioinformatics team, Turku Centre for Biotechnology, Tykistökatu 6, FI-20521 Turku, Finland

## Abstract

Most northern insect species experience a period of developmental arrest, diapause, which enables them to survive over the winter and postpone reproduction until favorable conditions. We studied the timing of reproductive diapause and its long-term effects on the cold tolerance of *Drosophila montana*, *D. littoralis* and *D. ezoana* females in seasonally varying environmental conditions. At the same time we traced expression levels of 219 genes in *D. montana* using a custom-made microarray. We show that the seasonal switch to reproductive diapause occurs over a short time period, and that overwintering in reproductive diapause has long-lasting effects on cold tolerance. Some genes, such as *Hsc70*, *Jon25Bi* and *period*, were upregulated throughout the diapause, while others, including *regucalcin*, *couch potato* and *Thor*, were upregulated only at its specific phases. Some of the expression patterns induced during the sensitive stage, when the females either enter diapause or not, remained induced regardless of the later conditions. qPCR analyses confirmed the findings of the microarray analysis in *D. montana* and revealed similar gene expression changes in *D. littoralis* and *D. ezoana*. The present study helps to achieve a better understanding of the genetic regulation of diapause and of the plasticity of seasonal responses in general.

Seasonally changing environmental conditions present various kinds of challenges for species living at high latitudes, setting requirements for the plasticity of life-history traits important to reproduction and survival. Accordingly, many northern species from insects to vertebrates are able to forecast the forthcoming adverse season on the basis of annual changes in environmental cues and to use this information to optimally time their development and reproduction. While biochemical and physiological responses can usually be reversed over a short time scale, the traits showing developmental plasticity, like reproductive diapause, tend to require a longer time to be terminated^1^. In photoperiodically induced adult reproductive diapause, females’ ovarian development is halted to a pre-vitellogenic stage during late summer, continuing only when the days become longer and warmer in spring. This kind of winter diapause is usually accompanied by physiological, morphological and behavioural changes that allow insects to cope with various kinds of stresses during the cold period^2^. Tracing the expression kinetics, i.e. changes in the expression patterns of genes associated with diapause, does not only offer a unique opportunity to gain information on the genetic regulation of the diapause itself, but also offers preliminary steps to identify some of the candidate genes underlying the adaptation to northern environments.

Daily and seasonal changes in life-history traits important in adaptation to changing environmental conditions have usually been studied at more or less constant light:dark and/or temperature cycles in the laboratory^3^,^4^. While studies like these are extremely helpful for detecting responses to specific environmental cues and tracing linkages between phenotypic changes and gene expression, they may not reveal the complete roles of daily and seasonal rhythms in wild populations. In studies that involve simultaneous changes in daily temperature and lighting gradients, the entrainments caused by these factors have been found to reinforce each other and narrow down e.g. the timing of activity peaks of insects^5^,^6^. For example, Vanin *et al.*^7^ studied the activity rhythms and accompanying changes in the expression levels of specific clock genes in *D. melanogaster* flies in the wild. Interestingly, their study shows that several key laboratory-based assumptions about circadian behaviour in this species, including the anticipation of lights on and off transitions, the midday siesta, and the dominance of light stimuli over temperature, are not supported by observations in naturally fluctuating conditions^7^.

Seasonal rhythms have been studied in natural surroundings less often than daily rhythms even though these kinds of studies would provide valuable information about adaptation to seasonality. Vesala *et al.*^8^ studied the cold tolerance of *D. montana* flies by mimicking the seasonal changes in day length and temperature of the flies’ collection site, and showed an increase in the cold tolerance of the flies towards the winter to be accompanied by changes at specific metabolite levels. This kind of study scheme can also be applied to study the environmental factors evoking facultative reproductive diapause, as well as to trace phenotypic and transcriptional changes during different phases of diapause, which should be understood as *a process* rather than *a status*^9^. In a facultative reproductive diapause, the diapause induction usually takes place at a pre-diapause stage as a response to shortening day length and decreasing temperature. During the initiation phase, the intensity of reproductive diapause quickly reaches its maximum and is accompanied by a rapid decrease in the insects’ metabolic rate, while the maintenance phase is characterized by maintaining the developmental arrest and a relatively low metabolic rate. The termination phase is characterized by the start of the sexual maturation^9^. The studies by Rinehart *et al.*^10^ and Ragland *et al.*^11^ on the flesh fly, *Sarcophaga crassipalpis,* have elegantly shown how specific genes are up- or down-regulated in diapausing females compared to non-diapausing ones during different phases of diapause. However, these studies were performed in constant conditions in the laboratory, and they lack the information about gene expression kinetics when the flies shift from one diapause phase to another in changing environmental conditions.

In the present study we have traced the effects of seasonally changing conditions on the development (reproductive diapause vs. sexual maturation) and cold tolerance of the females of three sympatric northern *D. virilis* group species (*D. montana*, *D. littoralis* and *D. ezoana*), and simultaneously studied their gene expression kinetics. Females of all these species enter diapause when the day length decreases below a critical point, and can remain in this state for up to nine months^12^,^13^,^14^. However, these species differ in their seasonal life cycles^15^ and geographical distribution^16^. In this study, we aimed to find answers to the following questions: (i) when does the onset of diapause occur in an environment showing gradual changes both in day length and temperature, (ii) does the overwintering state of the females affect their fitness in terms of cold tolerance after the diapause has terminated, and (iii) how do the gene expression patterns of the studied set of 219 genes vary during the initiation, maintenance and termination phases of diapause in different seasonal environments.

## Results and discussion

**Figure 1:**
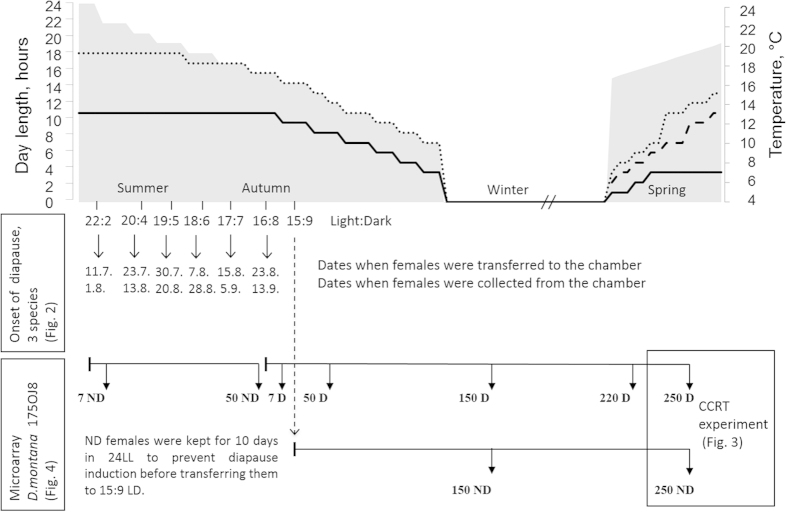
Light:dark cycles and temperature conditions in the environmental chamber and sample collection points. The grey area (scale on left Y-axis) shows seasonal changes in the day length and the lines (scale on the right Y-axis) respective changes in temperature during the day (dashed line), dawn/dusk in spring (thicker dashed line) and the night (solid line). The light:dark cycles and the dates indicate when the females were transferred into the chamber and when the different age ND (non-diapause) and D (diapause) females were collected. In addition to the microarray strain (175OJ8, *D. montana*), two additional *D. montana* strains and three *D. littoralis* and *D. ezoana* strains were collected for detecting the onset of reproductive diapause, and for qPCR (samples 150D and 150 ND) and CCRT experiments (samples 250D and 250ND).

**Figure 2:**
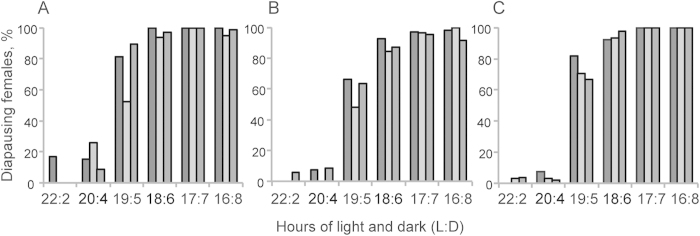
Onset of reproductive diapause. Seasonal switch to reproductive diapause in A) *D. montana* (strains 3OL8, 26OL8 and 175OJ8), B) *D. littoralis* (strains 202OJ8, 219J8 and 280OJ8) and C) *D. ezoana* (strains 67OJ8, 124OJ8 and 143OJ8) females (order of the strains as listed here). The proportion of diapausing females increased sharply in all three species in photoperiods shorter than 20:4LD. The critical day length (CDL), where the proportion of the females entering to reproductive diapause exceeded 50%, was 19:5LD in all species and strains.

**Figure 3:**
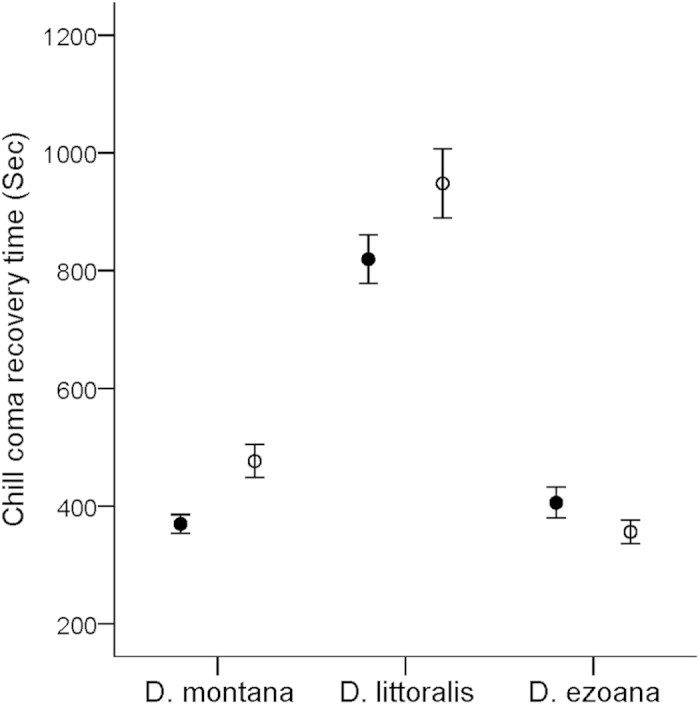
Chill coma recovery times of overwintered females. Chill coma recovery times (CCRT; seconds ± SE) of 250-day-old *D. montana*, *D. littoralis* and *D. ezoana* females that had overwintered in the climate chamber in reproductive diapause (filled circles) or in non-diapausing state (open circles). At the time of testing diapause was terminated and all females had fully developed ovaries.

**Figure 4:**
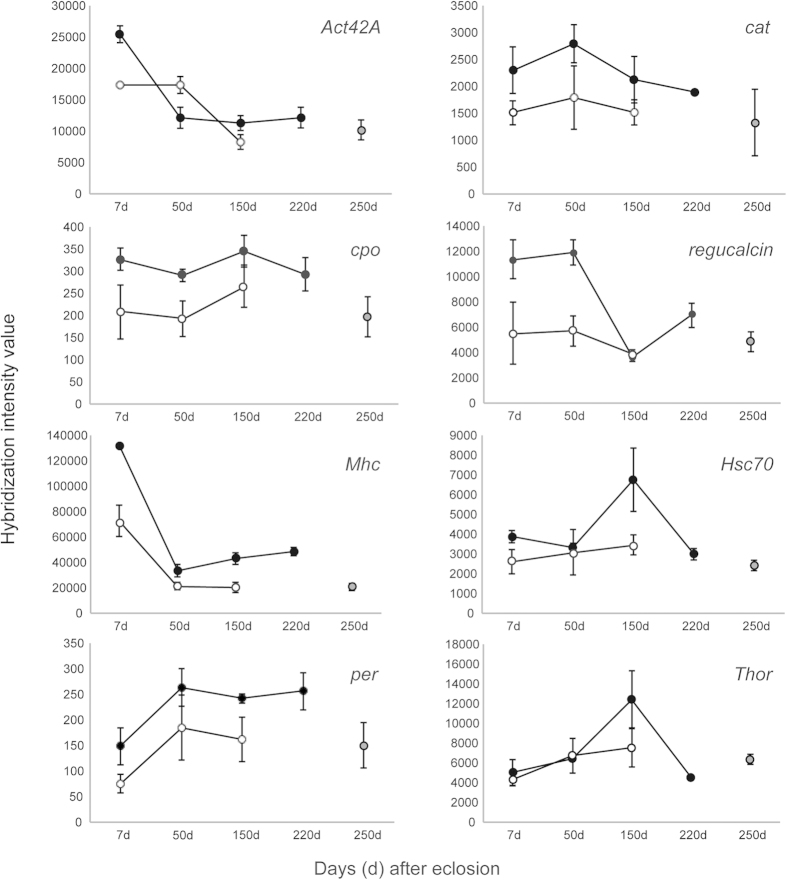
Seasonal gene expression kinetics. Seasonal gene expression kinetics is shown as means and standard error of means (SEM) of the hybridization intensity values among the microarray probes with the highest intensities for a given gene. Diapausing females are marked with filled circles, non-diapausing females with open circles, and the 250-day-old females that had overwintered in reproductive diapause, but had already terminated reproductive diapause and developed ovaries, are marked separately with gray.

Rearing the females of three northern *Drosophila virilis* group species in a climate chamber mimicking seasonal changes at their home site (Fig. 1) revealed several interesting phenomena linked with diapause and seasonal adaptation. First, the seasonal switch to reproductive diapause occurred in all three species at approximately the same day length as has been found to occur in *D. montana* in constant light:dark cycle and temperature conditions in the laboratory^14^,^17^ (Fig. 2). Second, in *D. montana* and *D. littoralis* the overwintering state of the females appeared to affect their cold tolerance even after the diapause had terminated (Fig. 3). Third, changes in the expression level of the studied genes between the initiation, maintenance and termination phases of diapause, as well as between the females overwintering at diapause or non-diapause state, showed that the upregulation of most of the studied genes was phase specific, while a few genes that were upregulated during the early adulthood remained upregulated throughout the females’ lifespan (Table 1, Supplementary Table 1, Fig. 4).

### Seasonal switch to reproductive diapause occurs within a narrow time period

Seasonal timing of reproductive diapause was determined by checking the ovarian development stage of three weeks old *D. montana*, *D. littoralis* and *D. ezoana* females, which had been transferred into the climate chamber between July and August (see Fig. 1). The critical day length for diapause induction (CDL; 50% of females enter diapause) was found to be LD 19:5 (corresponding to July 30^th^) in all study species; after this date the proportion of diapausing females increased to nearly 100% within one week (Fig. 2). In previous studies the CDLs of *D. montana* strains from Oulanka population have been found to vary between LD 18.5:5.5 and LD 19:5 at 16 °C^14^,^17^. These findings support each other and confirm the argument by Lumme^12^ and Watabe^13^ that the reproductive diapause of *D. virilis* group flies is regulated mainly by photoperiodic cues.

### Females’ reproductive state during overwintering affects their cold tolerance after diapause has been terminated

The chill coma recovery times (CCRTs) of 250-day-old *D. montana*, *D. littoralis* and *D. ezoana* females were measured 30 days after the winter period had ended (Fig. 1), when also the females that had overwintered in diapause had mature ovaries. The recovery times of the females varied significantly both among the species (ANOVA: F_6,237_ = 2.183, P < 0.05) and between the females’ overwintering states (ANOVA: F_1,237_ = 5.477, P < 0.05). *D. montana* and *D. ezoana* females recovered equally fast (in average 6–7 min.; Tukey HSD, P = 0.077; Fig. 3), while *D. littoralis* females recovered more slowly (in 13–16 minutes) than *D. montana* (Tukey HSD, P < 0.001) or *D. ezoana* (Tukey HSD, P < 0.001). All three species are found in northern Scandinavia, where the winters are cold, but *D. littoralis* is distributed also in central and southern Europe^16^,^18^. The large distribution area of *D. littoralis* might have caused trade-offs in the cold and heat tolerance of the flies, leading to a lower level of cold tolerance^19^. However, it should be kept in mind that low temperature causes several types of injuries in insects, and accordingly, different types of protective mechanisms are needed^20^. CCRT test used here describes mainly tolerance to direct injuries that are caused by severe short-term cold exposure^20^, while the tolerance to indirect chilling injuries that is caused by milder cold exposure over longer period may be equally or even more vital^21^. Overall, the CCRTs of the flies of all study species were quite short, indicating high cold tolerance compared to those of most other Drosophila species^22^. Kellerman *et al.*^21^ showed (using Critical Thermal Minimum method) that in genus Drosophila the high cold tolerance is mostly restricted to two species groups; *D. virilis* (including the species used in this study) and *D. obscura*.

Vesala and Hoikkala^23^ have shown that diapausing *D. montana* flies recover faster from chill coma than non-diapausing flies if the flies are not acclimated to cold, and Vesala *et al.*^24^ have demonstrated that the CCRT of diapausing females are shortest between late autumn and early spring. Our study shows that the females’ overwintering state has even longer-lasting effects on their cold tolerance. We also found significant interaction between the species and the overwintering state of the females (ANOVA: F_2,237_ = 4.688, P < 0.01): while *D. montana* and *D. littoralis* females that had overwintered in diapause recovered faster from chill coma than the females that had overwintered with mature ovaries in (F_1,97_ = 10.388, P < 0.01 and F_1,87_ = 4.316, P < 0.01, respectively), there were no significant differences between the two overwintering types in *D. ezoana* (F_1,112_ = 1.117, P = 0.293, Fig. 3). Increased cold tolerance of *D. montana* and *D. littoralis* females that overwintered in diapause could be due to that some diapause-related mechanisms are still ‘switched on’ after the diapause has terminated and/or that these females are in general in a better condition in spring than those overwintering with mature ovaries.

### Gene expression kinetics during different phases of diapause

Gene expression changes that occur during diapause have been commonly studied by comparing the expression levels of diapausing and non-diapausing females maintained in constant diapause inducing or preventing light:dark cycles^25^,^26^,^27^. Moreover, most of the earlier studies have focused on gene expression changes occurring during a specific phase of diapause^26^,^28^, while only some studies have covered the whole diapause period^11^. We traced the gene expression kinetics during different phases of diapause and also between diapausing (D) and non-diapausing (ND) females.

### *Onset/initiation phase.* 

Initiation phase of diapause was studied by comparing the gene expression patterns of 7-day-old females that were destined to diapause and females that were at the early stage of sexual maturation (7D vs. 7ND; Fig. 1; Supplementary Table 1.). The gene showing highest upregulation in young diapause-destined females was *CG9747*, a gene with acyl-CoA Delta11-desaturase activity that is involved in lipid metabolism and oxidation-reduction processes^29^. Other genes upregulated in diapause-destined females included the cytoskeleton modification gene *Actin 42A (Act42A)*, circadian clock gene *period* (*per*) and *couch potato (cpo)*, which has been connected to diapause in *D. melanogaster*^30^ and in *Culex pipiens*^31^, as well as a calcium-binding protein *regucalcin* (homolog of *Drosophila cold acclimation* gene, *Dca*) (Fig. 4). All these genes, except *Act42A*, have been linked with the maintenance phase of diapause or with cold tolerance in *D. montana* in our earlier studies^24^,^26^,^32^,^33^.

To disentangle which genes are upregulated during the onset phase of diapause in contrast to the maintenance phase, we compared 7-day-old diapausing females to 50-day-old diapausing females. This comparison brought up multiple genes connected e.g. to phototransduction (Supplementary Table 1.) Upregulation of phototrasduction genes highlights the genetic basis of the response to diapause inducing day lengths in northern Drosophila species. Among these genes *neither inactivation nor afterpotential D (ninaD)* showed the highest upregulation in 7-day-old females and another highly expressed gene was *Jonah 25Bi* (*Jon25Bi)*, which is known to be involved in proteolysis and to have endopeptidase activity^29^. Also *Act42A* and *Myosin heavy chain (Mhc)* genes were upregulated in young diapausing females when compared to older females.

### *Maintenance phase.* 

As the flies aged and proceeded from the onset phase to the maintenance phase, we observed moderately low gene expression changes (FC < 2) in most of the studied genes. A comparison between the 50 (50D) and the 7 (7D) days old diapausing females revealed upregulation in four ribosomal genes, several heat shock and stress related genes, as well as genes linked to locomotion and circadian rhythms (Supplementary Table 1). As the flies aged further and the maintenance phase was prolonged to the overwintering phase (150D) (constant darkness and +4 °C for four months), the highest expression change was found in *Dopa decarboxylase* (*Ddc*), which catalyzes the final step in the synthesis of neurotransmitters dopamine and serotonin^34^. These so called biogenic amines have been connected to multiple phenomena in *Drosophila sp.*, including stress resistance^35^ and longevity^36^. Other upregulated genes in 150D females included several heat shock genes, of which many are known to be highly upregulated in several insect species during diapause^10^, and genes that are connected to circadian clock (*vrille* (*vri)* and *fruitless* (*fru*)).

### *Termination phase.* 

The termination of the reproductive diapause began at the end of the artificial year, during spring conditions. 5–10 females were removed from the chamber at different time points in the spring and their ovaries were checked to observe the initiation of the termination phase (data not shown). During the spring, the 220-day-old females had just initiated the termination phase of reproductive diapause (220D) and were still at the pre-vitellogenic stage. The highest expression differences in 220-day-old females compared to 150-day-old diapausing females were detected in the heat shock gene *Hsc70*, followed by the *Phosphogluconate mutase (Pgm)*, *inactivation no afterpotential D (InaD)* and *Arrestin 2 (Arr2)* genes which are all linked to phototransduction. This could indicate the importance of photoperiodic cues during the termination phase of the reproductive diapause, although it has been hypothesized that temperature would be the main cue in the termination of diapause^13^.

At the age of 250 days during the spring conditions, females’ diapause was fully terminated and all the females had mature ovaries. Comparison between samples 250D and 220D revealed drastic expression changes in many genes that had not been detected during other diapause phases. This is not surprising as diapause termination induces ovarian maturation and several physiological changes. Due to the large amount of differentially expressed genes between these two offset phases, only part of the data will be discussed here, and are not included in the Supplementary Table 1. Genes showing the highest upregulation in 250-day-old vitellogenic females, when compared to 220-day-old pre-vitellogenic females, included *Pyrroline 5-carboxylate reductase* (*P5cr*: FC 315.7), involved in proline biosynthesis, and three heat shock genes (*Hsp26*, *Hsp70/Hsp90 organizing protein homolog (Hop*) and *Hsp23*), and *Jon25Bi*.

### Environmental conditions experienced during early adulthood affect gene expression levels later in life

Salminen and Hoikkala^37^ have shown that the sensitive period of the onset of reproductive diapause in *D. montana* is after eclosion, and its length is affected by temperature and it is induced by photoperiodic cues. The environmental cues experienced during the early stages of life can potentially cause irreversible changes in development both within and between generations^38^. In here, we have studied the effect of early stage cues on within generation gene expression patterns between diapausing and non-diapausing females.

Comparisons between females that overwintered either in diapause (150D) or with mature ovaries (150ND) enabled us to identify gene expression differences between the females that were of the same age and that had been maintained in the same conditions after the first 10 days of their life (the first 10 days after eclosion were spent either in diapause inducing or diapause inhibiting conditions). Dissection of the ovaries showed that females of the 150D group were in diapause while the females of the 150ND group had not laid their eggs or absorbed the yolk from the eggs during the overwintering period (i.e. they still had fully developed ovaries). Comparison of the gene expression patterns between 150D and 150ND females (Table 1) revealed upregulation in several genes in diapausing females, including *Catalase* (*Cat)*, *Act42A*, *per*, *Mhc*, and *Thor* (Fig. 4), suggesting that these gene have an important role in overwintering diapausing females.

Interestingly, many of the genes upregulated in the earlier phases of diapause were also upregulated in 150D females when compared to 150ND females, even though these two groups had been maintained in the same conditions for more than 140 days and had been collected from the chamber during the same seasonal time point (overwintering; +4 °C and constant darkness). The same trend was also seen in the non-diapausing 150-day-old females, which showed a high resemblance to the younger non-diapausing females (data not shown). These findings show that the switch before sexual maturation to reproductive diapause during the first 10 days of their life leads to the establishment of semi-permanent gene expression profiles that persist throughout adulthood. The findings also emphasize the importance of the correct timing of diapause and the long lasting effects of the induced developmental pathways later in life (Table 1, Fig. 4).

### Gene expression changes between different phases of diapause

Our study pinpoints various genes whose function is connected to specific phases of diapause, but also those genes that are upregulated throughout the diapause. From the 219 genes on the microarray, *Act42A*, *Mhc*, *Jon25Bi*, *Thor*, *per*, *CG6785*, *draper (drpr), Ecdysone-induced protein 28/29kD (Eip71CD)* and *Hsc70* (Table 1), stayed upregulated during different phases of diapause (Fig. 4).

Actin and myosin proteins are involved in the structural constituent of cytoskeleton and may be involved in the modification of the insects’ cytoskeleton during early adulthood rather than in diapause regulation per se. However, actin has been found to be highly abundant during the early diapause in *Nasonia vitripennis* larvae^39^ and *Culex pipiens* adults^40^. In our study, both *Act42A* and *Mhc* genes were upregulated in young females (7D) during the onset stage of diapause, as well as in overwintering diapausing females (150D), when compared to non-diapausing overwintering females (150ND). *Jon25Bi* also known as *Serine protease 4*, has a serine-type endopeptidase activity. Upregulation of *Jonah*-genes has been detected in *D. melanogaster* immediately after diapause, during egg development, and the gene has been suggested to play a role in post-diapause regulation of digestive activity^41^, as well as in the signal-transduction activating Toll-pathway in immune response^42^. In our study, *Jon25Bi* was upregulated throughout the onset, maintenance and termination periods of diapause (Table 1). *Thor* (synonyms: *elF4E binding protein*, d*4E-BP*), on the other hand, was upregulated during the maintenance (50D) and overwintering (150D) stages of diapause. In *Drosophila*, *Thor* is regulated at the transcriptional level by a forkhead transcription factor Foxo^43^,^44^, which is a downstream signaling component of the insulin/IGF1 pathway^45^. Shutdown of the insulin-signaling pathway, leading to Foxo activation, has been suggested to play a role in the induction of reproductive diapause in *Culex pipiens*^46^. The peak in the expression level of *Thor* in diapausing *D. montana* females during the maintenance and overwintering period may thus be a reflection of increased activity of Foxo. *Thor* transcription has also been connected to starvation and oxidative stress in *Drosophila*^47^,^48^ and we have found it to be upregulated during cold acclimation in non-diapausing *D. montana* females^24^. The circadian clock gene *period* (*per*) has been found to show dramatic changes in its expression level in the presence or absence of a thermoperiod and/or photoperiod^49^,^50^. Interestingly, *per* was upregulated throughout the life-cycle of diapausing *D. montana* females including the overwintering period (150D females in constant temperature and darkness). Ikeno *et al.*^51^ have detected the involvement of *per* in diapause induction also in bean bug, *Riptortus pedestris*, where silencing of *per* through RNAi caused the bugs to avert diapause even when they were raised in a diapause-inducing photoperiod.

### Consistency of gene expression patterns between different *D. montana* strains and sister species

**Figure 5:**
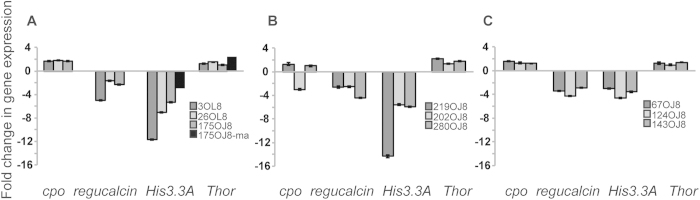
Gene expression levels between the three studied Drosophila species. Microarray results were validated from the *D. montana* strain 175OJ8 with qPCR. Beside the validation, expression levels of four candidate genes were compared between 150-day-old diapausing and non-diapausing females in two additional *D. montana* (**A**), three *D. littoralis* (**B**) and three *D. ezoana* (**C**) isofemale strains. Fold change differences of the *Histone 3.3A* and *Thor* genes in *D. montana* strain 175OJ8 used in the microarray study are shown in *D. montana* figure. The two other genes, *cpo* and *regucalcin*, did not show significant expression changes in the microarray study. Significance levels together with fold change values are given in the supplementary table 2 for all the comparisons.

Gene expression patterns of *His3.3A*, *regucalcin*, *cpo* and *Thor* genes were investigated with a qPCR method using 150-day-old diapausing and non-diapausing *D. montana* females from the strain that was used in the microarray assay and two additional strains. In addition, the expression of the same genes was observed from three strains of *D. littoralis* and *D. ezoana* (Fig. 5, Table 3 in supplementary material). For these studies one upregulated (*Thor*) and one downregulated (*His3.3A*) gene (in 150-day-old diapausing flies) were selected, as well as two genes (*cpo* and *regucalcin*) that did not show significant differences between diapausing and non-diapausing females in the microarray study, but have been connected to diapause in our previous studies in *D. montana*^26^,^32^,^33^.

qPCR results showed similar up- and downregulation as observed in the microarray assay for all the genes studied, and species comparisons indicated a consistency in gene expression between them; the only exception was *cpo*, which showed opposite patterns of gene expression in *D. montana* and *D. ezoana,* compared to one *D. littoralis* strain (Fig. 5). The highest overall expression values were observed in *His3.3A,* with significant downregulation in diapausing females in all three species, confirming the observation in the *D. montana* microarray study. Histone genes have been found to be downregulated during diapause also in *N. vitripennis*^39^ and *Caenorhabditis elegans*^52^ and the expression of these genes is known to be connected with DNA replication and protein translation^53^. Among the other three genes, *Thor* was significantly upregulated in diapausing *D. montana* females in the microarray assay, while in the qPCR analysis the expression changes reached significant levels in only one *D. montana* strain and in one of the two *D. ezoana* and *D. littoralis* strains (Fig. 5). In the microarray assay, the variance in the expression of *regucalcin* gene between the three biological replicates of the 150ND females was so large that it is hard to say whether the gene was up- or downregulated in comparison to 150D females. In the qPCR study, however, *regucalcin* was significantly downregulated in 150D females in all the three species. Finally, *cpo* showed a slight, but non-significant upregulation in 150D females in the microarray assay, while in qPCR a significant upregulation was evident in all the *D. montana* strains and in one *D. ezoana* strain, and surprisingly a significant downregulation in one *D. littoralis* strain.

## Conclusions

Gene expression and phenotypic changes induced by photoperiodic and/or temperature cues play an important role in adaptation to seasonally varying environments and are a widespread phenomenon among temperate species. However, modulation of gene expression, which is one of the main mechanisms leading to plasticity in life-history traits, has only seldom been studied in conditions resembling those in the wild. We determined the onset of reproductive diapause in three northern sympatric *D. virilis* group species, showing that the seasonal time window for entering to diapause is almost identical between these sympatric species, indicating strong adaptation in the timing of reproductive diapause. When the gene expression kinetics was studied between the different phases of diapause in *D. montana*, the elevated role of the phototransduction genes in the onset phase indicated the important role of the photoperiodic cues on the correct responses at the physiological level. Most importantly, we also show that some of the studied genes whose upregulation was induced at the onset phase of diapause, were upregulated throughout the lifespan of the females when compared to non-diapausing females experiencing the same environmental conditions. These findings highlight the importance of entering reproductive diapause at a correct seasonal time and its long lasting effects on the flies’ life-history traits, and enable us to plan and carry out new studies on flies’ diapause and cold tolerance e.g. on the whole transcriptome level.

## Materials and methods

### Study species and strains

The study was performed using adult females from three isofemale lines of *Drosophila montana* (3OL8, 26OL8 and 175OJ8), *D. littoralis* (202OJ8, 218OJ8 and 280OJ8) and *D. ezoana* (67OJ8, 124OJ8 and 143OJ8). The microarray experiment was performed using *D. montana* strain 175OJ8, while the phenotypic studies (switch to diapause and changes in cold tolerance) and qPCR were performed with the females of all nine strains. The strains were established from the progenies of females collected from Oulanka (Finland; 66 °N and 29 °E) in summer 2008 and maintained in the laboratory under diapause-preventing conditions (continuous light, 19 ± 1 °C, 65% humidity) since their establishment. Experiments were started within one year of the strains’ establishment.

### Mimicking environmental conditions in a climate chamber

*D. montana*, *D. littoralis* and *D. ezoana* females were collected from the maintenance stock bottles within one day of eclosion and transferred into the climate chamber (Sanyo MLR-351H, Sanyo, CA, USA), which had been programmed to mimic daily and seasonal changes in day length and temperature from early summer to late spring at the flies’ home site in northern Finland. Transferring young females into the chamber in early and late summer conditions enabled us to obtain both non-diapausing and diapausing females, which were then reared in the chamber for up to 9 months (Fig. 1). Humidity was kept at 60% throughout the artificial year, except during the winter period when it was decreased to 30%. Daily cycles in photoperiod and temperature were changed weekly at the same pace as these changes occur in nature; the only exception was the overwintering period during which the flies were maintained in constant darkness and at +4 °C for four months. This period was kept shorter and milder than the winter in the wild, which enabled us to keep both diapausing (D) and non-diapausing (ND) females alive over the winter period. (Fig. 1).

### Seasonal timing of reproductive diapause

The day length at which *D. montana*, *D. littoralis* and *D. ezoana* females enter reproductive diapause was traced by transferring freshly emerged females of three isofemale strains (50–80 females per strain) per species into the chamber during six time points representing the conditions between 11th of July (LD 22:2) and 23^rd^ of August (LD 16:8) in the wild. The ovarian developmental stage of the females was checked 21 days after the females had been transferred to the chamber (Fig. 1), using a protocol described in Tyukmaeva *et al.*^14^.

### Cold tolerance of overwintered *D. montana*, *D. littoralis* and *D. ezoana* females

The effects of overwintering on the cold tolerance of 250-day-old female flies that had spent the winter either in diapause (250D) or with mature ovaries (250ND), was estimated by measuring their chill coma recovery times (CCRT)^54^. Samples of 250ND females for these experiments were obtained by maintaining a group of freshly emerged females from each strain for ten days after their eclosion in constant light and at 19 °C to induce ovarian development, and transferring them into the chamber in early September conditions (3^rd^ of September). Samples of 250D females were obtained by transferring a set of females that had emerged on the same day as the above-mentioned ND females and transferring them immediately into the chamber (23^rd^ of August) in diapause inducing conditions (Fig. 1). The cold tolerance of overwintered ND and D females was determined with the CCRT test at the age of 250 days. At this stage, the termination period of the diapause of 250D females was over, and the females from both groups had mature ovaries.

CCRTs of the females was measured by keeping them at −6 °C for 16 hours^24^, and placing them after this on dishes with individual compartments for each fly at 21 ± 1 °C. CCRT was measured as the time (to the nearest second) it took for the females to recover from chill coma (stand up), i.e. to regain the lost membrane potential after a cold shock. The tests were performed on four subsequent days, using 15–20 females per strain per overwintering type (*D. montana* 175OJ8 and *D. littoralis* 219OJ8 strain had a slightly smaller sample size of the non-diapausing flies). The CCRTs were log-transformed to obtain equal variances. The effect of the overwintering state (diapausing or non-diapausing) on the CCRT was analysed using Analysis Of Variance (ANOVA) with CCRT as the dependent variable, and overwintering type, species and strain (nested within species) as factors. Replicate was included in the analysis as a random factor. Tukey’s HSD post-hoc test was applied to pair-wise comparisons between the species and ANOVA to compare the effect of overwintering state on recovery times separately in each species. Analyses were performed using IBM SPSS Statistics software 20.

### Collecting samples for studies on seasonal gene expression kinetics

Seasonal changes in females’ gene expression kinetics were traced using a custom-made microarray on ND and D females of *D. montana* strain 175OJ8 using three biological replicates. The conditions for rearing and sampling the females for these studies are shown in Fig. 1.

The females that had been transferred into the chamber in light and temperature conditions resembling those of 11^th^ of July at the collection site in Oulanka, Finland (long day conditions) started to develop ovaries, and samples of these females were collected from the chamber 7 (7ND sample) or 50 (50ND sample) days later. These samples represented non-diapausing individuals at an early and full stage of vitellogenesis, respectively.

Diapause-destined females, which had been transferred into the chamber on the 23^rd^ of August (short day conditions) were collected from the chamber during several time points (Fig. 1). The initial sample of females consisted of 7-day-old females that were at the initiation phase of diapause (7D). The second and third samples consisted of females that were at the maintenance phase of the diapause; the second sample consisted of 50-day-old diapausing females (50D) and the third sample of 150-day-old overwintering diapausing females (150D). These samples offered a possibility to trace changes in gene expression between different stages of diapause.

Differences in the gene expression levels between overwintering non-diapausing and diapausing females were studied using 150-day-old D and ND females that had been collected from the chamber during the winter conditions. These females had experienced the same environmental conditions since the early September (photoperiod 15:9 LD). The last two samples consisted of overwintered females that were at the termination phase of reproductive diapause. Sample 220D was collected 10 days, and sample 250D 30 days after the end of the winter period, and they consisted of 220-day-old females with pre-vitellogenic ovaries, and 250-day-old females with mature ovaries, respectively.

### Microarray analysis on *D. montana*

Flies were collected from the environmental chambers and immediately snap frozen with liquid nitrogen and kept at −84 °C until needed. Before the RNA extractions the flies were submerged into a pre-cooled (2 hours at −84 °C) RNAlaterICE solution (Applied Biosystems), kept overnight (∼16 hours) at −20 °C and dissected to check their ovarian development status (D vs. ND). Total RNA was extracted from two pooled females with Ambion RNAqueous 96 well total RNA Isolation Kit with DNase treatment (Qiagen) and purified with MinElute kit (Qiagen). The concentration and purity of RNA was measured with NanoDrop (NanoDrop Technologies, Wilmington, DE, USA) and the integrity with Bioanalyzer (Agilent). Microarray sample preparation prior to hybridization to the array was carried out as described in Kankare *et al.*^26^.

Seasonal changes in the expression levels of 219 genes were traced in *D. montana* females from the strain 175OJ8 using a custom designed DNA microarray constructed for this species^24^,^27^; complete list of genes on the array is presented in Supplementary Table 4). Microarray data was analyzed using R (R Foundation for Statistical Computing) and Bioconductor software^55^. The data were quantile normalized and the statistical analyses were carried out using Bioconductor’s Limma package and specific fold change (FC) and p-value cutoffs were applied for each comparison (minimum values of FC = 2, FDR = 0.05).

### qPCR studies: validation of the microarray results and checking the consistency of the gene expression patterns among the species

To validate the microarray results on *D. montana* strain 175OJ8, and to find out how they apply to the other study species and strains, we performed quantitative real time PCR (qPCR) with a sub set of genes for 150-day-old diapausing and non-diapausing for *D montana* (two additional strains), *D. littoralis* (three strains) and *D. ezoana* (three strains) females. For each strain we used five biological replicates and three technical replicates.

RNA for qPCR was extracted using flies that had been collected from the climate chamber at the same time as the microarray samples. Total RNA was extracted from two females per sample using a modified Tri-reagent (Ambion) protocol followed by a clean-up (RNeasy mini kit, Qiagen) and a DNAse (Qiagen) treatment. The purity and the integrity of RNA were measured as described above. cDNA was generated from the RNA with iScript Reverse Transcription Supermix (Bio-Rad Laboratories), following the manufacturer’s protocol and using RNA samples diluted to equal concentrations of 30 ng/μl.

The study genes chosen for qPCR analyses were *Histone3.3A* (*His3.3A*), *regucalcin*, *couch potato* (*cpo*) and *Thor. foraging* (*for*), *no receptor potential A* (*norpA*) and *Ribosomal protein L11* (*RpL11*) were selected as control genes as they showed least differences in their expression levels when comparing all the samples in the microarray data (including expression patterns of both diapausing and non-diapausing females).. Primers for the control- and experimental genes were designed using *D. montana* cDNA sequences^26^, and the NetPrimer program (http://www.premierbiosoft.com/netprimer/). Amplification efficiency was checked for each strain separately, using 2-fold serial dilutions of pooled cDNA (Supplementary Table 2). For qPCR analyses, 20 μl reactions were used containing 10 μl of 2x Power SYBR Green PCR Master Mix (Bio-Rad Laboratories), 0.3 μM of each gene-specific primer and 1 μl of cDNA solution. Cycling conditions in Bio-Rad CFX96 instrument were: 3 min. 95 °C, 10 s. 95 °C, 10 s. 55 °C and 30 s. 72 °C (40x), followed by melting curve analysis (65 °C–95 °C) for amplification specificity checking. Gene expression values for all comparisons were calculated with normalized expression method (∆∆(Ct)) using CFX96 Manager Software 2.0 (Bio-Rad Laboratories) with real efficiency values. Statistical significance of the results was computed with REST 2009 program (http://www.gene-quantification.de/rest-2009.html) using 10000 iterations and bootstrapping method.

## Additional Information

**How to cite this article**: Salminen, T. S. *et al.* Seasonal gene expression kinetics between diapause phases in *Drosophila virilis* group species and overwintering differences between diapausing and non-diapausing females. *Sci. Rep.* **5**, 11197; doi: 10.1038/srep11197 (2015).

## Supplementary Material

Supplementary Information

## Figures and Tables

**Table 1:** Gene expression fold changes between different diapause phases and seasons.

***Gene symbol***	**Initiation 7D / 7ND**	**Initiation 7D / 50D**	**Maintenance 50D / 7D**	**Overwintering 150D / 50D**	**Overwintering 150D / 150ND**	**Termination 1 220D / 150D**	**Termination 2 250D / 220D**	**Molecular function/biological process/protein function**
*Act42A*	2.5^**^	6.4^***^	—	—	2.5^**^	—	—	structural constituent of cytoskeleton
*Arr1*	—	—	—	—	3.1^**^	—	—	opsin binding, photoreceptor cell maintenance
*Cat*	—	—	—	—	2.9^**^	—	—	catalase activity
*CG6785*	—	—	9.4^***^	6.1^***^	4.0^**^	—	—	stearoyl-CoA 9-desaturase activity
*CG8630*	—	—	—	—	3.6^**^	—	—	voltage-gated potassium channel activity
*CG15531*	—	—	—	—	9.3^*^	—	—	response to heat
*CG17928*	—	—	—	—	10.8^***^	—	—	stearoyl-CoA 9-desaturase activity
*Droj2*	—	—	—	3.2^***^	3.2^***^	—	—	Wnt-protein binding, signal transduction
*drpr*	—	—	2.2^**^	—	2.2^**^	—	2.7^***^	locomotor
*dy*	—	—	—	2.1^*^	2.6^**^	—	—	imaginal disc-derived wing morphogenesis
*Eip71CD*	—	4.2^***^	—	—	2.9^*^	—	3.3^***^	determination of adult lifespan
*fru*	—	—	—	3.0^***^	2.2^**^	—	—	multi-organism reproductive process, mating
*Gale*	2.8^**^	2.5^***^	—	—	2.9^***^	—	—	UDP-glucose 4-epimerase activity
*Hsc70*	—	—	3.4^***^	3.3^***^	2.9^***^	13.0^**^	—	heat shock chaperonin-binding
*ken*	—	—	—	—	2.5^***^	—	—	female analia development
*LanA*	—	—	2.4^***^	—	5.0^***^	—	—	immune response; growth; locomotory behavior
*Mhc*	—	3.9^***^	—	—	2.2^**^	—	—	actin-dependent ATPase activity
*ninaD*	—	28.4^***^	—	—	3.1^*^	—	—	scavenger receptor activity, phototransduction
*per*	2.1^**^	—	2.7^***^	2.1^*^	2.2^*^	—	—	phosphoglycerate mutase activity
*so*	—	—	—	—	2.0^**^	—	—	chaperone binding, protein lipidation
*Thor*	—	—	2.3^**^	2.5^**^	2.4^**^	—	—	immune response, determination of adult lifespan
*vri*	—	—	—	4.0^***^	2.1^*^	—	—	circadian rhythm

List of genes that showed significant expression changes with Fold Change (FC) ≥ 2, when the 150-day-old diapausing and non-diapausing females were compared. Table also shows the other diapause phases, where the same genes were upregulated. Molecular function and/or biological process and /or protein function are according to the Flybase version 3_2013. Significance levels: ^*^P < 0.05 ^**^P < 0.01 ^***^P < 0.001.
