# Supplementary material for: Seasonal gene expression kinetics between diapause phases in Drosophila virilis group species and overwintering differences between diapausing and non-diapausing females
Source: Sci Rep. 2015 Jun 11;5:11197. doi: 10.1038/srep11197 (PMC4463020; doi:10.1038/srep11197)
Supplement: Supplementary Information [file srep11197-s1.doc]

**Supplementary information**

**Seasonal gene expression kinetics between diapause phases in *Drosophila virilis* group species and overwintering differences between diapausing and non-diapausing females**

Tiina S. Salminen^1,2^, Laura Vesala^1,2^, Asta Laiho^3^, Mikko Merisalo^1^, Anneli Hoikkala^1^ and Maaria Kankare^1^

^1^ University of Jyvaskyla, *Department of Biological and Environmental Science, P.O. Box 35, FI-40014 University of Jyväskylä, Finland*

^2^ *BioMediTech, Biokatu 6, F1-33014 University of Tampere, Finland*

^3^ *Finnish DNA Microarray Centre, Bioinformatics team, Turku Centre for Biotechnology, Tykistökatu 6, FI-20521 Turku, Finland*

Corresponding author:

PhD Tiina S. Salminen

BioMediTech, Biokatu 6, FI-33014 University of Tampere, Finland.

Email: tiina.s.salminen@uta.fi

Phone : +358 40 593 5734

**Supplementary table 1.** List of genes that showed significant expression changes with Fold Change (FCs) ≥ 2 during the initiation phase of diapause (7D vs. 7ND and 7D vs. 50D), maintenance phase (50D vs. 7D), during overwintering period (150D vs. 50D, and 150D vs. 150ND), early termination phase (220D vs. 150D) and later termination phase (250D vs. 220D). Molecular function and/or biological process and /or protein function are taken from the Flybase version 3_2013.

| **Gene symbol** | **FCs and significance levels of gene expression changes between different phases of diapause** | | | | | | |  |
| --- | --- | --- | --- | --- | --- | --- | --- | --- |
|  | **Initiation**  **7D / 7ND** | **Initiation**  **7D / 50D** | **Maintenance**  **50D / 7D** | **Overwintering**  **150D / 50D** | **Overwintering**  **150D / 150ND** | **Termination 1**  **220D / 150D** | **Termination 2**  **250D / 220D** | **Molecular function/biological process/protein function** |
| *Ace* | - | 2.1*** | - | - | - | 2.9** | 2.8*** | cholinesterase activity, phototaxis |
| *Acon* | - | 3.7*** |  | - | - | - | - | tricarboxylic acid cycle |
| *Act42A* | 2.5** | 6.4*** | - | - | 2.5** | - | - | structural constituent of cytoskeleton |
| *Adf1* | - | - | 3.3*** | - | - | - | 2.5*** | synapse assembly, locomotion |
| *Amph* | 2.3** | 2.1** | - | - | - | - | - | protein localization |
| *Arr1* | - | - | - | - | 3.1** | - | - | opsin binding, photoreceptor cell maintenance |
| *Arr2* | 2.4** | 2.2** | - | - | - | 6.1*** | - | opsin binding, photoreceptor cell maintenance |
| *Atp-alpha* | - | 4.6** |  | - | - | - | - | sodium:potassium-exchanging ATPase activity |
| *B4* | - | - | - | 2.5** | - | - | - | circadian rhythm |
| *Cat* | - | - | - | - | 2.9** | - | - | catalase activity |
| *CG5001* | - | 5.2*** | - | - | - | - | - | UDP-glucose 4-epimerase activity |
| *CG6785* | - | - | 9.4*** | 6.1*** | 4.0** | - | - | stearoyl-CoA 9-desaturase activity |
| *CG7182* | - | - | 4.0*** | 2.5*** | - | - | - | oxidoreductase activity, lateral inhibition |
| *CG8630* | - | - | - | - | 3.6** | - | - | voltage-gated potassium channel activity |
| *CG9747* | 5.0** | - | 6.7*** | - | - | - | - | protein-cysteine S-palmitoleyltransferase activity |
| *CG9934* | - | - | 2.5*** | - | - | - | - | unfolded protein binding, response to heat |
| *CG15531* | - | - | - | - | 9.3* | - | - | response to heat |
| *CG17928* | - | - | - | - | 10.8*** | - | - | stearoyl-CoA 9-desaturase activity |
| *CG34366* | - | 3.6*** | - | - | - | - | - | cyl-CoA delta11-desaturase activity |
| *CG42318* | - | 3.3*** | 2.0*** | - | - | 3.0*** | - | ubiquitin-ubiquitin ligase activity |
| *CkIIalfa* | - | - | 2.5*** | - | - | - | - | protein binding, rhythmic process |
| *Col* | - | 2.7** | - | - | - | - | - | cytochrome-c oxidase activity, sleep |
| *cpo* | 2.4** | - | - | - | - | - | - | mRNA binding, dormancy process |
| *cwo* | - | 3.9** |  | - | - | - | - | regulation of circadian rhythm |
| *dco* | - | - | 3.2** | - | - | - | - | kinase activity, response to stimulus |
| *Ddc* | - | - | - | 8.5** | - | - | - | aromatic-L-amino-acid decarboxylase activity |
| *desat1* | 2.5** | - | - | - | - | - | - | stearoyl-CoA 9-desaturase activity |
| *desat2* | - | 2.1** | - | - | - | 4.2** | - | stearoyl-CoA 9-desaturase activity |
| *Droj2* | - | - | - | 3.2*** | 3.2*** | - | - | Wnt-protein binding, signal transduction |
| *drl* | - | 2.7*** | - | - | - | - | - | heat shock protein binding, neurogenesis |
| *drpr* | - | - | 2.2** | - | 2.2** | - | 2.7*** | locomotor |
| *dy* | - | - | - | 2.1* | 2.6** | - | - | imaginal disc-derived wing morphogenesis |
| *Ef1alfa48D* | - | 2.2** | 3.8*** | - | - | - | - | determination of adult lifespan |
| *Eip71CD* | - | 4.2*** | - | - | 2.9* | - | 3.3*** | determination of adult lifespan |
| *FKBP59* | - | - | 2.5*** | - | - | - | - | protein binding, detection of light stimulus |
| *Fmr1* | - | - | 2.8** | - | - | - | - | protein binding, rhythmic process |
| *for* | - | - | 3.4*** | - | - | - | - | cGMP-dependent protein kinase activity |
| *fru* | - | - | - | 3.0*** | 2.2** | - | - | multi-organism reproductive process, mating |
| *Gale* | 2.8** | 2.5*** | - | - | 2.9*** | - | - | UDP-glucose 4-epimerase activity |
| *Gapdh* | - | 4.2*** | - | - | - | - | 2.9*** | glycolysis |
| *Gbeta76C* | - | 3.8*** | - | - | - | - | - | phototransduction |
| *gl* | - | 3.1** | - | - | - | - | - | entrainment of circadian clock |
| *Hop* | - | - | 3.4*** | - | - | - | 54.2*** | unfolded protein binding, protein folding |
| *Hsc70* | - | - | 3.4*** | 3.3*** | 2.9*** | 13.0** | - | heat shock chaperonin-binding |
| *Hsc70Cb* | - | - | 3.1*** | - | - | - | - | chaperone binding, protein folding |
| *Hsf* | - | - | 2.6** | - | - | - | - | protein binding, response to heat |
| *Hsp60* | - | - | 2.2*** | - | - | - | - | unfolded protein binding, response to heat |
| *Hsp68* | - | - | 2.3*** | 2.1* | - | - | - | protein lipidation |
| *Hsp83* | - | - | 3.1*** | - | - | - | - | determination of adult lifespan |
| *Hsp67Bc* | 3.2** | - | - | - | - | - | 12.1*** | ATPase activity, response to cold |
| *Hsr omega* | - | - | 2.8*** | 2.7** | - | - | - | protein localization |
| *imd* | 2.7*** | 6.8*** | - | - | - | - | - | protein binding, immune response |
| *inaD* | - | 2.6** | - | - | - | 3.3** | - | photoreceptor activity, phototransduction |
| *Jon25Bi* | - | 27.5*** | - | 3.6* | - | 7.4** | 24.6*** | proteolysis, immune response |
| *ken* | - | - | - | - | 2.5*** | - | - | female analia development |
| *LanA* | - | - | 2.4*** | - | 5.0*** | - | - | immune response; growth; locomotory behavior |
| *Madm* | - | - | 2.9*** | - | - | - | - | protein serine/threonine kinase activity |
| *Mekk1* | - | - | 3.3*** | - | - | - | - | MAP kinase activity, response to stress |
| *Mhc* | - | 3.9*** | - | - | 2.2** | - | - | actin-dependent ATPase activity |
| *nan* | 2.2** | - | - | - | - | - | - | calcium channel activity |
| *neb* | - | - | - | - | - | 28.8 | - | calcium channel activity |
| *NinaC* | - | 2.3** | - | - | - | - | - | protein ser/threo kinase activity, phototransd. |
| *ninaD* | - | 28.4*** | - | - | 3.1* | - | - | scavenger receptor activity, phototransduction |
| *numb* | - | - | 2.1** | - | - | - | - | protein binding, regulation of devel. process |
| *Pgm* | - | 6.6*** | - | - | - | 2.8** | - | elongation factor-2 kinase activity |
| *PEK* | - | - | 3.8** | - | - | - | - | protein binding, rhythmic process |
| *per* | 2.1** | - | 2.7*** | 2.1* | 2.2* | - | - | phosphoglycerate mutase activity |
| *Prp5* | - | - | 3.3*** | - | - | - | - | regulation of alternative mRNA splicing |
| *regucalcin* | 2.8** | 2.2** | - | - | - | - | - | multicellular organism reproduction, SMP-30 |
| *Rh6* | 2.5** | - | - | - | - | - | - | phototransduction |
| *Rop* | - | - | 2.6*** | - | - | - | - | response to light stimulus |
| *RpL11* | - | - | 2.1*** | - | - | - | - | Ribosomal protein |
| *RpL19* | - | - | 2.3*** | - | - | - | - | Ribosomal protein |
| *RpL27A* | - | 2.8** | 2.2*** | - | - | - | - | Ribosomal protein |
| *RpL40* | - | 2.4** | 2.5*** | - | - | - | - | Ribosomal protein, Ubiquitin |
| *rut* | 2.3** | - | - | 3.8*** | - | - | 5.1*** | response to stress |
| *sas* | 2.3** | 3.0*** | - | - | - | 3.4*** | - | glycosylation |
| *sisA* | 2.5** | 4.7*** | - | - | - | - | - | protein binding, sex determination |
| *slmb* | - | - | 2.6** | - | - | - | 2.6*** | phosphoprotein binding, rhythmic process |
| *sls* | - | 9.3*** | - | 2.6* | - | - | - | actin binding, locomotion |
| *spin* | - | - | - | 6.9* | - | - | - | protein binding, entrainment of circadian clock |
| *stc* | - | - | 2.8*** | - | - | - | - | biological regulation |
| *stv* | - | - | 2.1*** | - | - | - | - | regulation of transcription |
| *so* | - | - | - | - | 2.0** | - | - | chaperone binding, protein lipidation |
| *Thor* | - | - | 2.3** | 2.5** | 2.4** | - | - | immune response, determination of adult lifespan |
| *tilB* | - | - | 3.6*** | - | - | - | - | temperature compensation of the circadian clock |
| *Tpi* | - | 3.4*** | - | - | - | - | - | determination of adult lifespan |
| *tra* | - | - | 2.4** | - | - | - | 3.2*** | protein binding, female sex determination |
| *trpl* | 4.1** | - | - | - | - | - | - | protein binding;, phototransduction |
| *tutl* | - | - | - | 2.4* | - | - | - | adult locomotory behavior |
| *Ubi-p63e* | - | 3.1** | 3.0*** | - | - | - | - | ubiquitin |
| *vri* | - | - | - | 4.0*** | 2.1* | - | - | circadian rhythm |
| *y* | 3.2** | 21.9*** | - | - | - | - | - | melanin biosynthetic process |

Significance levels: * P < 0,05 ** P < 0,01 *** P < 0,001

**Supplementary Table 2.** Genes and primers used in the qPCR analysis. Experimental genes are marked with “e” and control genes with “c”. Efficiency values (E%) and correlation coefficient values (R^2^) are given for all the species.

Gene F/R primer sequence (5’ – 3’) *D. montana D. ezoana D. littoralis*

E% R^2^ E% R^2^ E% R^2^

*His3.3A* (e) GGCACCCCGCAAACAATT 99.1 0.999 91.9 0.997 96.3 0.999

GACTTCTGGTAACGACGAAT

*regucalcin* (e) ATTGTCAACTGGGACGGTGT 94.7 0.999 100.4 0.999 88.2 0.999

CTCTTGACCACCGACACATT

*cpo* (e) GCATCGGCTGCTCCATTA 96.7 0.991 107.0 0.992 93.2 0.980

TTATGGCATCATCCGCTA

*Thor* (e) GGCACCAAGGTGATCTATGAA 95.0 0.985 94.0 0.997 91.9 0.996

TGCTCCTGTTCCTCGATTTT

*for* (c) GCTTGAGGAGACGCACTATCA 86.2 0.999 94.0 0.998 90.1 0.995

TCACGCCTTCGGGAGACTCAC

*NorpA* (c) GGAGACCAAAGAGAATGA 91.4 0.992 105.8 0.982 104.0 0.973

AATCACGAATGTCGCTCAC

*RpL11* (c) GCAGCCCGTGTTTTCTAAGG 101.4 0.999 95.3 0.991 104.8 0.995

TACTCCCGAACTTTCAAGCCAC

**Supplementary table 3.** qPCR validation of the microarray results comparing the expression level differences of four candidate genes between 150-day-old diapausing and non-diapausing females in three isofemale strain from *D. montana*, *D. ezoana* and *D. littoralis.*

Gene *D. montana D. ezoana D. littoralis*

Strain 26OL8 3OL8 175OJ8 67OJ8 124OJ8 143OJ8 202OJ8 219OJ8 280OJ8

Upregulated genes

*cpo* 1.8*** 1.7* 1.7*** 1.6* 1.3^NS^ 1.2^NS^ 3.0***^1)^ 1.3^NS^ 1.0^NS^

*Thor* 1.5** 1.2^NS^ 1.0^NS^ 1.2^NS#)^ 1.6^NS^ 1.4 ^NS^ 1.4^NS^ 2.2* 1.8**

Downregulated genes

*His 3.3A* 7.1*** 11.7*** 5.3*** 3.0*** 4.6*** 3.6*** 5.6*** 14.3*** 5.9***

*regucalcin* 1.7*** 5.0*** 2.3*** 3.4*** 4.3*** 2.9*** 2.5** 2.6*** 4.4***

^1)^ downregulated.

Statistical significance: NS not significant, *P < 0.05 **P < 0.01 ***P < 0.001

**Supplementary table 4.** List of all the genes in the custom made DNA microarray for *Drosophila montana*.

| **No** | **Gene ID** | **CG number** | **Gene name/synonym** | **FBgn** |
| --- | --- | --- | --- | --- |
| 1 | *5-HT7* | 12073 | *Serotonin receptor 7* | FBgn0004573 |
| 2 | *14-3-3-epsilon* | 31196 | *14-3-3ε* | FBgn0020238 |
| 3 | *Ace* | 17907 | *Acetylcholine esterase* | FBgn0000024 |
| 4 | *Acon* | 9244 | *Aconitase* | FBgn0010100 |
| 5 | *Act_42A* | 12051 | *Actin 42A* | FBgn0000043 |
| 6 | *Adar* | 12598 | *Adenosine deaminase acting on RNA* | FBgn0026086 |
| 7 | *Adf1* | 15845 | *Adh transcription factor 1* | FBgn0000054 |
| 8 | *Adh* | 3481 | *Alcohol dehydrogenase* | FBgn0000055 |
| 9 | *amd* | 10501 | *α methyl dopa-resistant* | FBgn0000075 |
| 10 | *Amph* | 8604 | *Amphiphysin* | FBgn0027356 |
| 11 | *Ankyrin* | 1651 | *Ankyrin* | FBgn0011747 |
| 12 | *ari-1* | 5659 | *ariadne* | FBgn0017418 |
| 13 | *Arr1* | 5711 | *Arrestin 1* | FBgn0000120 |
| 14 | *Arr2* | 5962 | *Arrestin 2* | FBgn0000121 |
| 15 | *ato* | 7508 | *atonal* | FBgn0010433 |
| 16 | *Atp-alpha* | 5670 | *Na pump α subunit* | FBgn0002921 |
| 17 | *b* | 7811 | *black* | FBgn0000153 |
| 18 | *B4* | 9239 | *B4* | FBgn0023407 |
| 19 | *bsk* | 5680 | *basket* | FBgn0000229 |
| 20 | *Btk29A* | 8049 | *Btk family kinase at 29A* | FBgn0003502 |
| 21 | *btv* | 15148 | *beethoven* | FBgn0023096 |
| 22 | *cac* | 1522 | *cacophony* | FBgn0005563 |
| 23 | *Caki* | 6703 | *Calcium/calmodulin-dependent protein kinase* | FBgn0013759 |
| 24 | *Cam* | 8472 | *Calmodulin* | FBgn0000253 |
| 25 | *CaMKII* | 18069 | *Calsium/calmodulin-depend.prot.kin.II* | FBgn0004624 |
| 26 | *Cat* | 6871 | *Catalase* | FBgn0000261 |
| 27 | *Cdc37* | 12019 | *Cdc37* | FBgn0011573 |
| 28 | *Cdk9* | 5197 | *Cyclin-dependent kinase 9* | FBgn0019949 |
| 29 | *CdsA* | 7962 | *CDP diglyceride synthetase* | FBgn0010350 |
| 30 | *CG3061* | 3061 | *CG3061* | FBgn0038195 |
| 31 | *CG3814* | 3814 | *CG3814* | FBgn0025692 |
| 32 | *CG4049* | 4049 | *CG4049* | FBgn0034976 |
| 33 | *CG4164* | 4164 | *CG4164* | FBgn0031256 |
| 34 | *CG5001* | 5001 | *Heat shock prot.cognate DnaJ, N-term.* | FBgn0031322 |
| 35 | *CG6785* | 6785 | *CG6785* | FBgn0032399 |
| 36 | *CG7182* | 7182 | *Heat shock protein 70 (Hsp70)* | FBgn0035878 |
| 37 | *CG7650* | 7650 | *CG7650* | FBgn0036519 |
| 38 | *CG8630* | 8630 | *CG8630* | FBgn0038130 |
| 39 | *CG9743* | 9743 | *CG9743* | FBgn0039756 |
| 40 | *CG9747* | 9747 | *CG9747* | FBgn0039754 |
| 41 | *CG9934* | 9934 | *CG9934* | FBgn0032467 |
| 42 | *CG10990* | 10990 | *CG10990* | FBgn0030520 |
| 43 | *CG11251* | 11251 | *CG11251* | FBgn0036346 |
| 44 | *CG12030* | 12030 | *CG12030* | FBgn0035147 |
| 45 | *CG12020* | 12020 | *Heat shock prot.cognate DnaJ, C-term.* | FBgn0035273 |
| 46 | *CG14650* | 14650 | *Heat shock prot.cognate DnaJ, N-term.* | FBgn0037252 |
| 47 | *CG15531* | 15531 | *CG15531* | FBgn0039755 |
| 48 | *CG17928* | 17928 | *CG17928* | FBgn0032603 |
| 49 | *CG34366* | 34366 | *CG34366* | FBgn0085395 |
| 50 | *CG42318* | 42318 | *CG42318* | FBgn0260941 |
| 51 | *CkIIalfa* | 17520 | *Casein kinase II aplha subunit* | FBgn0000258 |
| 52 | *Clk* | 7391 | *Clock* | FBgn0023076 |
| 53 | *CoI* | 34067 | *Mitochondrial Cytochrome c oxidase I* | FBgn0013674 |
| 54 | *cpo* | 31243 | *couch potato* | FBgn0000363 |
| 55 | *CrebB17A* | 6103 | *Cyclic-AMP resp.el.bind.prot. B17A* | FBgn0014467 |
| 56 | *cry* | 3772 | *cryptochrome* | FBgn0025680 |
| 57 | *Csp* | 3695 | *Cysteine string protein* | FBgn0004179 |
| 58 | *cwo* | 17100 | *clockwork orange* | FBgn0259938 |
| 59 | *cyc* | 8727 | *cycle* | FBgn0023094 |
| 60 | *Cyt-b5-r* | 13279 | *Cytochrome b5-related* | FBgn0000406 |
| 61 | *dare* | 12390 | *defective in the avoidance of repellents* | FBgn0015582 |
| 62 | *Dat* | 3318 | *Dopamine N acetyltransferase* | FBgn0019643 |
| 63 | *dco* | 2048 | *discs overgrown/double time* | FBgn0002413 |
| 64 | *Ddc* | 10697 | *Dopa decarboxylase* | FBgn0000422 |
| 65 | *desat1* | 5887 | *desat1* | FBgn0086687 |
| 66 | *desat2* | 5925 | *desat2* | FBgn0043043 |
| 67 | *disco* | 9908 | *disconnected* | FBgn0000459 |
| 68 | *DnaJ-1* | 10578 | *DnaJ-like-1* | FBgn0015657 |
| 69 | *dnc* | 32498 | *dunce* | FBgn0000479 |
| 70 | *Dr* | 1897 | *Drop* | FBgn0000492 |
| 71 | *drd* | 33968 | *drop dead, beltless* | FBgn0260006 |
| 72 | *drl* | 17348 | *derailed* | FBgn0015380 |
| 73 | *Droj2* | 8863 | *DnaJ-like-2* | FBgn0038145 |
| 74 | *drpr* | 2086 | *draper* | FBgn0027594 |
| 75 | *dsf* | 9019 | *dissatisfaction* | FBgn0015381 |
| 76 | *Dsor1* | 15793 | *Downstream of raf1* | FBgn0010269 |
| 77 | *dy* | 9355 | *dusky* | FBgn0004511 |
| 78 | *e* | 3331 | *ebony* | FBgn0000527 |
| 79 | *eag* | 10952 | *ether a go-go* | FBgn0000535 |
| 80 | *Ef1alfa48D* | 8280 | *Elongation factor 1 alpha 48D* | FBgn0000556 |
| 81 | *eIF-4a* | 9075 | *Eukaryotic initiation factor 4a* | FBgn0001942 |
| 82 | *Eip71CD* | 7266 | *Ecdysone-induced protein 28/29kD* | FBgn0000565 |
| 83 | *ey* | 1464 | *eyeless* | FBgn0005558 |
| 84 | *FKBP59* | 4535 | *FK506-binding protein FKBP59* | FBgn0029174 |
| 85 | *Fmr1* | 6203 | *Fmr1* | FBgn0028734 |
| 86 | *for* | 10033 | *foraging* | FBgn0000721 |
| 87 | *fru* | 14307 | *fruitless* | FBgn0004652 |
| 88 | *Fst* | 9434 | *Frost* | FBgn0037724 |
| 89 | *Gad1* | 14994 | *Glutamic acid decarboxylase 1* | FBgn0004516 |
| 90 | *Gapdh1* | 12055 | *Glyceraldehyde 3 phosp.dehydrogen. 1* | FBgn0001091 |
| 91 | *Gapdh2* | 8893 | *Glyceraldehyde 3 phosp.dehydrogen. 2* | FBgn0001092 |
| 92 | *Gbeta76C* | 8770 | *G protein beta subunit 76C* | FBgn0004623 |
| 93 | *gl* | 7672 | *glass* | FBgn0004618 |
| 94 | *Gp93* | 5520 | *Glycoprotein 93* | FBgn0039562 |
| 95 | *Gr66A* | 7189 | *Gustatory receptor 66a* | FBgn0035870 |
| 96 | *G-salpha60A* | 2835 | *G protein sα 60A* | FBgn0001123 |
| 97 | *hang* | 32575 | *hangover* | FBgn0026575 |
| 98 | *Histone_H3.3A* | 5825 | *Histone H3.3A* | FBgn0014857 |
| 99 | *hiw* | 32592 | *highwire* | FBgn0030600 |
| 100 | *homer* | 11324 | *homer* | FBgn0025777 |
| 101 | *Hop* | 2720 | *Hsp70/Hsp90 organizing protein hom.* | FBgn0024352 |
| 102 | *Hsc70-2* | 7756 | *Heat shock protein cognate 2* | FBgn0001217 |
| 103 | *Hsc70-3* | 4147 | *Heat shock protein cognate 3* | FBgn0001218 |
| 104 | *Hsc70-4* | 4264 | *Heat shock protein cognate 4* | FBgn0001219 |
| 105 | *Hsc70-5* | 8542 | *Heat shock protein cognate 5* | FBgn0001220 |
| 106 | *Hsc70Cb* | 6603 | *Hsc70Cb* | FBgn0026418 |
| 107 | *Hsf* | 5748 | *Heat shock factor* | FBgn0001222 |
| 108 | *Hsp20* | 4461 | *Hsp20/CG4461* | FBgn0035982 |
| 109 | *Hsp23* | 4463 | *Heat shock protein 23* | FBgn0001224 |
| 110 | *Hsp26* | 4183 | *Heat shock protein 26* | FBgn0001225 |
| 111 | *Hsp60* | 12101 | *Heat shock protein 60* | FBgn0015245 |
| 112 | *Hsp60B* | 2830 | *Heat shock protein 60 related* | FBgn0011244 |
| 113 | *Hsp67Bc* | 4190 | *Heat shock gene 67Bc* | FBgn0001229 |
| 114 | *Hsp68* | 5463 | *Heat shock protein 68* | FBgn0001230 |
| 115 | *Hsp83* | 1242 | *Heat shock protein 83* | FBgn0001233 |
| 116 | *Hsr_omega* | 31400 | *Heat shock RNA ω* | FBgn0001234 |
| 117 | *ilp7* | 13317 | *Insulin-like peptide 7* | FBgn0044046 |
| 118 | *imd* | 5576 | *immune deficiency* | FBgn0013983 |
| 119 | *inaC* | 6518 | *inactivation no afterpotential C* | FBgn0004784 |
| 120 | *inaD* | 3504 | *inactivation no afterpotential D* | FBgn0001263 |
| 121 | *inaF* | 2457 | *inactivation no afterpotential F* | FBgn0260812 |
| 122 | *InR* | 18402 | *Insulin-like receptor* | FBgn0013984 |
| 123 | *ix* | 13201 | *intersex* | FBgn0001276 |
| 124 | *jet* | 8873 | *jetlag* | FBgn0031652 |
| 125 | *Jon25Bi* | 8867 | *Jonah 25Bi* | FBgn0020906 |
| 126 | *Jon66Cii* | 7170 | *Jonah 66Cii* | FBgn0035887 |
| 127 | *ken* | 5575 | *ken and barbie* | FBgn0011236 |
| 128 | *knk* | 6217 | *knickkopf* | FBgn0001321 |
| 129 | *l(2)efl* | 4533 | *lethal (2) essential for life* | FBgn0011296 |
| 130 | *LanA* | 10236 | *Laminin A* | FBgn0002526 |
| 131 | *lark* | 8597 | *lark* | FBgn0011640 |
| 132 | *Madm* | 1098 | *MLF1-adaptor molecule* | FBgn0027497 |
| 133 | *MAPk-Ak2* | 3086 | *MAP kinase activated protein-kinase-2* | FBgn0013987 |
| 134 | *Mekk1* | 7717 | *Mekk1* | FBgn0024329 |
| 135 | *Mhc* | 17927 | *Myosin heavy chain* | FBgn0086783 |
| 136 | *mle* | 11680 | *maleless* | FBgn0002774 |
| 137 | *mnb* | 42273 | *minibrain* | FBgn0259168 |
| 138 | *Mpk2* | 5475 | *Mpk2* | FBgn0015765 |
| 139 | *na* | 1517 | *narrow abdomen* | FBgn0002917 |
| 140 | *nan* | 5842 | *nanchung* | FBgn0036414 |
| 141 | *neb* | 10718 | *nebbish* | FBgn0004374 |
| 142 | *Nf1* | 8318 | *Neurofibromin 1* | FBgn0015269 |
| 143 | *ninaC* | 5125 | *neither inactivation nor afterpotential C* | FBgn0002938 |
| 144 | *ninaD* | 31783 | *neither inactivation nor afterpotential D* | FBgn0002939 |
| 145 | *ninaE* | 4550 | *neither inactivation nor afterpotential E* | FBgn0002940 |
| 146 | *nompA* | 13207 | *no mechanoreceptor potential A* | FBgn0016047 |
| 147 | *nonA* | 4211 | *no on or off transient A* | FBgn0004227 |
| 148 | *norpA* | 3620 | *no receptor potential A* | FBgn0262738 |
| 149 | *numb* | 3779 | *numb* | FBgn0002973 |
| 150 | *ogre* | 3039 | *optic ganglion reduced* | FBgn0004646 |
| 151 | *Ork1* | 1615 | *Open rectifier K^+^ channel 1* | FBgn0017561 |
| 152 | *ort* | 7411 | *ora transientless* | FBgn0003011 |
| 153 | *p38b* | 7393 | *p38b* | FBgn0024846 |
| 154 | *P5cr* | 6009 | *Pyrroline 5-carboxylate reductase* | FBgn0015781 |
| 155 | *para* | 9907 | *paralytic* | FBgn0260993 |
| 156 | *PEK* | 2087 | *Pancreatic eIF-2aplha kinase* | FBgn0037327 |
| 157 | *per* | 2647 | *period* | FBgn0003068 |
| 158 | *Pgm* | 5165 | *Phosphogluconate mutase* | FBgn0003076 |
| 159 | *Pi3K92E* | 4141 | *Pi3K92E* | FBgn0015279 |
| 160 | *Pka-C1* | 4379 | *cAMP-dependent protein kinase 1* | FBgn0000273 |
| 161 | *Pka-R2* | 15862 | *cAMP-dependent protein kinase R2* | FBgn0022382 |
| 162 | *ple* | 10118 | *pale* | FBgn0005626 |
| 163 | *Pp1-87B* | 5650 | *Protein phosphatase 1 at 87B* | FBgn0004103 |
| 164 | *Prp5* | 6227 | *CG6227* | FBgn0030631 |
| 165 | *pwn* | 11101 | *pawn* | FBgn0003174 |
| 166 | *qtc* | 14039 | *quick-to-court* | FBgn0028572 |
| 167 | *rdgB* | 11111 | *retinal degeneration B* | FBgn0003218 |
| 168 | *regucalcin* | 1803 | *regucalcin* | FBgn0030362 |
| 169 | *Rev1* | 12189 | *Rev1* | FBgn0035150 |
| 170 | *Rh3* | 10888 | *Rhodopsin 3* | FBgn0003249 |
| 171 | *Rh4* | 9668 | *Rhodopsin 4* | FBgn0003250 |
| 172 | *Rh5* | 5279 | *Rhodopsin 5* | FBgn0014019 |
| 173 | *Rh6* | 5192 | *Rhodopsin 6* | FBgn0019940 |
| 174 | *Rop* | 15811 | *Ras opposite* | FBgn0004574 |
| 175 | *RpL11* | 7726 | *Ribosomal protein L11* | FBgn0013325 |
| 176 | *RpL19* | 2746 | *Ribosomal protein L19* | FBgn0002607 |
| 177 | *RpL27A* | 15442 | *Ribosomal protein L27A* | FBgn0261606 |
| 178 | *RpL40* | 2960 | *Ribosomal protein L40* | FBgn0003941 |
| 179 | *rut* | 9533 | *rutabaga* | FBgn0003301 |
| 180 | *Sas* | 5232 | *Sialic acid phosphate synthase* | FBgn0038045 |
| 181 | *sbb* | 5580 | *scribbler* | FBgn0010575 |
| 182 | *scb* | 8095 | *scab* | FBgn0003328 |
| 183 | *Sdc* | 10497 | *Syndecan* | FBgn0010415 |
| 184 | *serT* | 4545 | *Serotonin transporter* | FBgn0010414 |
| 185 | *sgg* | 2621 | *shaggy* | FBgn0003371 |
| 186 | *Sh* | 12348 | *Shaker* | FBgn0003380 |
| 187 | *shakB* | 34358 | *shaking B* | FBgn0085387 |
| 188 | *shi* | 18102 | *shibire* | FBgn0003392 |
| 189 | *sisA* | 1641 | *sisterless A* | FBgn0003411 |
| 190 | *slgA* | 1417 | *sluggish A* | FBgn0003423 |
| 191 | *slmb* | 3412 | *supernumerary limbs* | FBgn0023423 |
| 192 | *slo* | 10693 | *slowpoke* | FBgn0003429 |
| 193 | *Slob* | 6772 | *Slowpoke binding protein* | FBgn0024290 |
| 194 | *sls* | 1915 | *sallimus* | FBgn0086906 |
| 195 | *SNF4Agamma* | 17299 | *SNF4/AMP-activated protein kinase gamma subunit* | FBgn0025803 |
| 196 | *so* | 11121 | *sine oculis* | FBgn0003460 |
| 197 | *Sod* | 11793 | *Superoxide dismutase* | FBgn0003462 |
| 198 | *spin* | 8428 | *spinster* | FBgn0086676 |
| 199 | *stc* | 3647 | *shuttle craft* | FBgn0001978 |
| 200 | *stv* | 32130 | *starvin* | FBgn0086708 |
| 201 | *syt* | 3139 | *synaptotagmin* | FBgn0004242 |
| 202 | *Thor* | 8846 | *Thor* | FBgn0261560 |
| 203 | *tilB* | 14620 | *touch insensitive larva B* | FBgn0014395 |
| 204 | *tim* | 3234 | *timeless* | FBgn0014396 |
| 205 | *tipE* | 1232 | *temperature-induced paralytic E* | FBgn0003710 |
| 206 | *tko* | 7925 | *technical knockout* | FBgn0003714 |
| 207 | *Tpi* | 2171 | *Triose phosphate isomerase* | FBgn0086355 |
| 208 | *tra* | 16724 | *transformer* | FBgn0003741 |
| 209 | *Treh* | 9364 | *Trehalase* | FBgn0003748 |
| 210 | *Trf* | 7562 | *TBP-related factor* | FBgn0010287 |
| 211 | *Trh* | 9122 | *Tryptophan hydroxylase* | FBgn0035187 |
| 212 | *trp* | 7875 | *transient receptor potential* | FBgn0003861 |
| 213 | *trpgamma* | 5996 | *trpγ* | FBgn0032593 |
| 214 | *trpl* | 18345 | *trp-like* | FBgn0005614 |
| 215 | *tutl* | 15427 | *turtle* | FBgn0010473 |
| 216 | *tws* | 6235 | *twins* | FBgn0004889 |
| 217 | *Ubi-p63e* | 11624 | *Ubiquitin-63E* | FBgn0003943 |
| 218 | *vri* | 14029 | *vrille* | FBgn0016076 |
| 219 | *y* | 3757 | *yellow* | FBgn0004034 |
